# Annotations

**Published:** 1910-03-26

**Authors:** 


### ANNOTATIONS.

### Tropical Hygiene.

The London School of Tropical Medicine is to be congratulated upon starting its courses of public lectures on subjects connected with tropical medicine and hygiene. At present there is a demand for simple, clear, and definite instruction in the essentials of prophylaxis and hygiene which every European who has occasion to spend a portion of his existence in a tropical or semi-tropical climate should be acquainted with, and it seems to us that these lectures will supply that demand admirably. Professor Simpson's course was most successful and satisfactory, and the second lecturer, Dr. C. W. Daniels, Director of the London School of Tropical Medicine, has not limited the scope of his demonstrations to specific diseases or questions of hygiene, but has taken a broad view of the whole domain that may come under the definition of tropical medicine. The first lecture dealt with general conditions of life in tropical countries, with malaria and mosquitoes; the second with other insect-carried diseases and with general rules of prophylaxis; the third with food and drink impurities and contaminations, and with such important matters as cholera, typhoid, and dysentery, while the fourth lecture will be devoted to typically contagious diseases and parasitic affections. We are glad to learn that these lectures, which were given at convenient hours during the afternoon, have been well attended, and that there has been expressed a desire to have a continuation of these courses during the summer. The London School of Tropical Medicine can do a great deal of good by popularising the essentials of tropical hygiene, and this preliminary series of lectures is a step in the right direction.

### The Beit Bequest.

Only recently the first list of Research Fellows under the provisions of Mr. Beit's will was published in these columns, with the subjects which each of the appointed Fellows proposes to investigate (The Hospital, March 5, 1910, p. 648). Perhaps, naturally, it did not strike British readers that there should be anything strange in the selection of British workers for these endowments; but, also naturally, South Africa has a different and quite logical view point of its own. Mr. Beit's millions were made in South Africa, though it may be well to remember that they came as much from the pocket of the British investor as from the reefs and galleries of the Rand mines. Therefore, says the profession in South Africa—and probably the public holds the same view—it is right and proper to devote some part of the bequest to the solution of South African problems. The Transvaal Medical Society has accordingly memoralised the Colonial Secretary of the Transvaal in Pretoria and has pointed out to him twelve pressing matters, all suitable and ripe for research. Mr. Smuts lent a favourable ear to the deputation, and with his support and approval a copy of the memorial was forwarded to Lord Milner. The latter is far too large-minded a man to allow himself to be influenced in connection with this appeal by the treatment he has received in South Africa, and it may not unreasonably be expected that the next list of Beit Fellows may contain names which will please the Transvaal Medical Society. The twelve problems which were selected as most urgent are: Typhoid and paratyphoid fever, including the question of carriers; pneumonia and pneumococcal infections; cerebro-spinal meningitis; rheumatism and heart disease, and the influence of altitude on health; tuberculosis and leprosy; African dysentery and liver abscess; bilharziosis; ankylostomiasis; Beri-beri, scurvy, and pellagra; malaria and blackwater fever; conditions of natives in kraals in regard to food values of diets; and the therapeutic value of South African flora. Some of these are not especially African problems; and some of them are already fairly well investigated, though we should be the last to assert that the final word has been said on any one of them.

### Urinalysis at the Bedside.

An ingenious method by which urine can be tested for albumin and sugar out of the resources of an ordinary household has lately been described by an American physician, Dr. Richter, of St. Louis. These tests are not merely within the compass of such improvised apparatus, but are also said to be quite delicate and to give most satisfactory results. They are therefore well worth bearing in mind, for the practitioner suddenly confronted with a case of coma or of convulsions may be very materially helped in his diagnosis and his treatment if he can decide at once whether the urine contains sugar or albumin. To test for the latter, take an ordinary spoon; half fill it with urine, add a pinch of salt, and heat it over a spirit lamp, or even a match. When it begins to steam and bubble, add a few drops of vinegar. This test is said to be more delicate than Heller's nitric acid method, and the white precipitate produced has the additional advantage of not discolouring the urine, as strong nitric acid very often does. Sugar is almost equally simple to test for. Dilute two or three drops of urine with a few drops of water in a teaspoon, and evaporate carefully to dryness by gentle heat. On slowly heating further, a characteristic orange-brown spot and the odour of burnt sugar or caramel will prove the presence of sugar; three parts per thousand of glucose can thus be detected with certainty. Urine containing no sugar leaves a smoky black residue with a urinous odour, but one quite distinct from that of burnt sugar. If more than 1 per cent. of sugar is present, a characteristic lump of charcoal is formed. These simple tests may be recommended to the attention of the profession, especially of those members who practise in remote country districts where chemists' shops are few and far between, and a patient seen in a state of coma may be many miles from the doctor's urine-testing case in his surgery.

